# Getting to effective housing policy for health: a thematic synthesis of policy development and implementation

**DOI:** 10.1080/23748834.2024.2328951

**Published:** 2024-04-04

**Authors:** Emily Nix, Andrew Ibbetson, Ke Zhou, Michael Davies, Paul Wilkinson, Ramona Ludolph, Helen Pineo

**Affiliations:** a Institute for Environmental Design and Engineering, University College London, London, UK; b Department of Public Health, Policy and Systems, University of Liverpool, London, UK; c Department of Public Health, Environments and Society, London School of Hygiene & Tropical Medicine, London, UK; d Department of Environment, Climate Change and Health, World Health Organization, Geneva, Switzerland; e Department of Urban Design and Planning, University of Washington, Seattle, WA, USA

**Keywords:** Housing, health, policy processes, review

## Abstract

Impacts of housing on health are well-recognised. Despite this, housing standards have been neglected and there are gaps in healthy housing policies, particularly in low and middle-income countries. Given the recent publication of the WHO Housing and health guidelines, and the need to implement these into policy at all scales, we carried out a focused search and thematic synthesis of available literature on the barriers and enablers to recent housing and health policy. We aimed to generate lessons of what works to support healthy housing policy development and implementation elsewhere. Twenty-three studies representing four countries were eligible for inclusion and covered housing-related risks of air quality, lead, accessible design, and housing conditions. Findings demonstrated that policy development and implementation were facilitated through awareness of housing and health, evidence of existing housing conditions and health impacts, collaborations across sectors and between residents and decision-makers and effective enforcement systems that employed incentives, tools such as certificates for compliance, and housing inspections. Concerns about economic viability and tensions between housing rights and responsibilities limited healthy housing policy for the ‘common good’. Despite limitations in the diversity of available evidence, this thematic synthesis provides a starting point for healthy and equitable housing for all.

## Introduction

Adequate housing is a human right (Office of the United Nations High Commissioner for Human Rights 2009). Housing can harm or support physical, mental and social health and well being through multiple pathways (Thomson *et al*. 2009). Poor quality housing can increase exposure to hot or cold indoor temperatures (Scovronick and Armstrong 2012) air pollutants (Shrubsole *et al*. 2012) toxic building materials (e.g. asbestos) and dampness and mould (Oreszczyn *et al*. 2006). Inadequate housing can further result in injuries from falls, fires, and electrocution and fail to protect against the ingress of disease vectors and infectious diseases (Office of the Deputy Prime Minister 2006, Nix *et al*. 2020), among other risks. In high-income settings, such as Europe, the environmental burden of disease from inadequate housing is substantial due to a multitude of risk factors (Braubach *et al*. 2011). For example, across 11 European countries, low indoor temperatures are estimated to result in 38 200 excess winter deaths per year, representing 12.8 excess deaths per 100 000 population (Braubach *et al*. 2011), and globally, 84,000 deaths from lung cancer were caused by residential radon in 2019 (Exchange GHD 2021). However, the environmental burden of disease is likely to be much greater in low- and middle-income countries (LMICs) (Haines *et al*. 2013) as housing quality tends to be lower due to high rates of informal housing development outside of regulations, often resulting in informal settlements (Ezeh *et al*. 2017). Well-designed and maintained housing that minimises or removes this negative impact on health is good for residents and beneficial for society (Braubach *et al*. 2011). Furthermore, improved housing can help achieve co-benefits for sustainability and planetary health, through energy efficiency measures that reduce carbon dioxide (CO2) emissions (Wilkinson *et al*. 2009, Howden-Chapman and Chapman 2012, Hamilton *et al*. 2015). Globally, residential buildings account of 22% of all energy consumption (the third biggest consumer after transport and other industries at 26% each) and 16% of all CO2 emissions, with demand continuing to rise (IEA (International Energy Agency 2021); thus, reductions across this sector are fundamental in meeting global climate targets. Housing is becoming an increasingly important policy area due to the global trends of urbanisation (Ezeh *et al*. 2017) the climate and energy crisis and population ageing that link to the safety, suitability, and provision of housing for all population groups. The provision of healthy housing is vital in supporting the achievement of the 2030 Sustainable Development Goals, particularly, Goal 11: Make cities and human settlements inclusive, safe, resilient, and sustainable (United Nations 2019).

Historically, the impacts of inadequate housing on health have been well recognised (Lopez 2009), with high rates of disease linked to ‘air-deprived’ tenements and lack of sanitation in the late 19^th^ century (Fairchild *et al*. 2010). Thousands of working people in Manchester, UK in 1845 were said to reside in *‘wretched, damp, filthy cottages, that the streets which surround them are usually in the most miserable and filthy condition, laid out without the slightest reference to ventilation’* (Smith *et al*. 2001). Similar housing conditions were reported in urban industrialised centres across Europe and North America during this period (Shaw 2004). Efforts to address housing conditions first centred on reforms which shifted responsibility for improvements from tenants to property owners (Blackmar 1995); however, lack of progress through incremental improvements later led to a policy shift to condemn entire districts of poor tenements (Lopez 2009). Eradication of entire settlements has been a common approach in recent decades to address informal housing in developing countries; however, this deeply contested practice was recognised as problematic by international agencies who have advocated for programmes that provide support to households to make incremental upgrades (Dupont 2008). From the 1990s, the self-help policy was considered unable to meet increasing housing demand – which led to policies that enabled favourable conditions for social actors (individuals, private companies, NGOs etc) to effectively provide affordable housing (Bredenoord and van Lindert 2010). Although, providing a more holistic approach to urban planning, focus has turned away from the housing question itself and largely failed to meet demand – with 2 billion people predicted to live in slums by 2030 (1/4 of the global population) (UN-Habitat 2003) – leading to agencies to advocate again for self-build initiatives with institutional, financial, and technical backing (Bredenoord and van Lindert 2010, UN-Habitat 2016).

Early housing codes included New York State’s First Tenement House Act (1867), which required fire escapes in each suite and a window in every room, the Second Tenement House Act (1879) closed a loophole and stipulated every habitable room required a window opening to plain air (Lopez 2009). This legislation was later replaced by Tenement Law of 1901 (or Veiller’s Law), which created the model for housing codes across the US (Fairchild *et al*. 2010). Tenement Laws fell out of favour in the US due a lack of enforcement and exemptions on existing building, until new housing advocates pushed for reform and Catherine Bauer’s book on Modern Housing ignited activism on housing, ultimately, leading to the 1937 Housing Act (Bauer 2020). More recently, in Europe, tendency towards privatisation and deregulation of housing has been linked to increased harms to health; for example, in the UK, an expanding private-rental market has been shown to have worse housing conditions compared to owner-occupied housing and deregulation of non-domestic building housing has been linked to lower housing for health (Clifford and Pineo 2023).

All that said, with decades of varying progress and the recognition of additional environmental health hazards there is a range of existing policy mechanisms that consider how housing design, materials, and operation influence health: Building regulations (or codes) can support healthy, safe, and accessible housing conditions, such as by requiring minimum ventilation rates to ensure appropriate air quality (Howieson *et al*. 2014) or to minimise radon levels (Denman *et al*. 2018). Regulations can prohibit the use of hazardous materials (such as lead and asbestos) (Magavern and Magavern 2018) ban tobacco smoking in the home (Hollar *et al*. 2017) or enforce the use of safety devices (e.g. fire alarms) (Harvey *et al*. 2013) to prevent or minimise harm. National or local authority policies and programmes can support the construction or refurbishment of healthy housing (Johnson 2016, Ambrose *et al*. 2018) such as professional training in accessible housing design and incentives for homeowners/landlords to make modifications to existing dwellings (CARE, ENGLAND R 2015). Other examples include policies that support vulnerable groups to access (Ong *et al*. 2015) or maintain adequate housing, such as fuel poverty payments to support energy use for home heating and cooling (Howden-Chapman *et al*. 2012).

Within the past decade, however, public health advocates have again called for renewed efforts to address substandard housing conditions and to update standards which have become neglected over time (Krieger and Higgins 2002, Werna *et al*. 2022). While housing policy has been recently significantly influenced by climate mitigation policies in developed countries (with recognition of health co-benefits), a recent examination of building regulations in England revealed a lack of public health evidence in the policy development process (Carmichael *et al*. 2020). Calls for renewed efforts are most keenly highlighted in the recent publication of the World Health Organization (WHO) Housing and health guidelines (HHGL) that provide evidence-based policy recommendations to promote health through housing design and management (World Health Organization 2018). Although limited in aspects (e.g. do not include evidence around vector-borne diseases), the WHO HHGL outline multiple risk factors in housing and provide evidence to enable health considerations in housing, energy, community development, and urban development policies.^1^ To realise health gains the guidelines need to be implemented within national, regional and local policy, to support this effort WHO commissioned a review of policy for healthy housing (World Health Organization 2021). This review found, rather unsurprisingly, that the existence, implementation and enforcement of adequate housing policy that appropriately considers health varies globally, with significant gaps, particularly in low and middle-income countries (World Health Organization 2021). Furthermore, numerous studies have demonstrated that the existence of good policy is not always sufficient and that implementation is influenced by a wide range of social, economic, political, and environmental factors (Carmichael *et al*. 2012, Pineo *et al*. 2020, Callway *et al*. 2020). Indeed, effective housing efforts are argued to depend on the capacity and resources of public agencies and to be limited by a lack of tenant power within a market-based system (Krieger and Higgins 2002). In low- and middle-income countries, housing development often follows informal construction practices and lack the enforceability of formal legal standards – leaving those most vulnerable to reside in inadequate conditions (Werna *et al*. 2022).

Given this call for renewed efforts to update housing standards and gaps in the existence of healthy housing policy globally, translation of the recently developed evidence-based WHO HHGL into local, regional, and national policy is vital to ensure universal access to adequate housing. This work aimed to understand the current barriers and enablers to housing and health policy across policy stages, from agenda setting to policy implementation. Understanding the relevant barriers and enablers can identify what worked, for whom and when, and guide the development and implementation of effective policy for healthy housing in additional settings. This paper further builds on the previously mentioned review of housing policy for health (World Health Organization 2021) and looks at studies that specifically discuss healthy housing policy development and implementation in relation to health risks outlined in the WHO HHGL.

## Methods

### Search scope and aims

This review builds on a broader study to identify policies, regulations, and legislation with the potential to promote healthy housing at (sub-) national levels (World Health Organization 2021). The study served as a basis for a policy repository and report, which will be key elements of the WHO healthy housing implementation toolkit. This paper focuses on studies that discuss the development and implementation of housing policies and builds on the findings presented in the report (World Health Organization 2021). The aim was to synthesise available evidence of healthy housing policy development and implementation. The specific objectives were to (a) identify the factors that enable or limit the development and implementation of healthy housing policy and (b) determine transferable lessons to support policy development and implementation in other settings, where policies are currently lacking or need strengthening. A wide range of policy approaches and instrument as shown Table 1 were considered as appropriate for this review, if they explicitly addressed a health-hazard outlined in the WHO HHGL.

**Table 1.** Housing policy approaches and instruments that can promote health, adapted from (World Health Organization 2021).

Broad policy approach	Specific instruments
Standards/regulations for healthy homes	Building regulation/standards Heat/cooling system regulation Mandated safety devices Prohibition of hazardous materials and substances Guidelines for air/water/radon levels Smoke-free housing policy Outdoor regulations Thermal comfort code
Constructing/retrofitting healthy housing	New housing construction programmes Refurbishment programmes Technical training Inspection and removal of hazards Clean energy programmes Water supply programmes Physical Subsidies/incentives for Modifications Land tax/planning policies
Ensuring access to healthy homes	Rehousing programmes Rental assistance Fuel poverty payments Clean fuel subsidies

This paper distinguishes between the processes of developing and implementing policy based on Howlett et al.’s (2016) description of policy-making, which combines the perspectives of several policy process theories (Howlett *et al*. 2016). Policy development includes agenda setting, formulation and decision-making (or adoption) and is characterised as a complex and often contested process of collaboration across diverse actors (Howlett *et al*. 2016). Policy implementation is similarly complex and involves enacting the policy, such as through the application of building regulations in a new development or banning tobacco smoking in social housing. Policy implementation should ideally be followed by evaluation. For the purposes of this study, we considered the stages of agenda setting, policy formulation, decision-making, and implementation. We did not consider papers evaluating the effectiveness of policy, e.g. papers reporting changes in health outcomes, as this was considered as a different type of evidence and beyond the scope of this review. Assessing effectiveness of policies on health outcomes would require a meta-analysis to pool effects and separate to the methods followed in this work.

The review used thematic synthesis, following the approach outlined by Thomas and Harden (2008) to understand how healthy housing policies are successfully developed and implemented, identifying specific barriers and enablers and producing an initial theory of what works, for whom and in which circumstances. Thematic synthesis has been proposed as a method to compile and integrate findings of qualitative data, as traditional systematic reviews often rely on quantitative data, such as from randomised control trials (RCTs) and synthesise findings through a meta-analysis (Thomas and Harden 2008). In this review, we directly analysed the text (as a form of qualitative data) from eligible papers, this included both quantitative and qualitative studies, as well as case studies that included expert opinion.

### Search strategy, information sources and checking of articles

The search strategy consisted of terms related to 1) housing policies, 2) health or housing conditions, and 3) health-risks related to housing as included in the WHO HHGL. Terms were identified by scanning key documents, the WHO HHGL and discussion with the review team. No language restrictions or study design filters were applied to the search strategy.

The following bibliographic databases were searched during November 2019: MEDLINE (via Ovid), EMBASE (via Ovid), Social policy and practice (via Ovid), Science Citation Index (via Web of Science), Political Science Database (via ProQuest), Scopus. The search strategy was tailored to each database; an example strategy is provided in the supplementary material. Search terms and date of search were documented in a research journal.

### Eligibility criteria

The eligibility criteria were based on the research aims. Research and case studies of any design were eligible, provided these following points: (i) reported substantive data on the development, adoption and/or implementation of healthy housing policy; (ii) the policy focus addressed a housing-related health risk as covered in the WHO HHGL. Papers that focused on a housing policy in which health or a health-risk was not an explicit focus were not included. For example, if the study focused relocation or housing construction policies, if health was not explicitly addressed, it was considered not relevant, although there may be health benefits from such policies. Papers that only described the policy details, and its impacts on health were considered outside the scope of this review, if it did not provide information on the development and implementation the policy.

Search results from all geographical regions and housing types and tenures (e.g. detached homes and flats; social and owner-occupier housing) were eligible for inclusion. Documents published before 2010 were excluded to focus on the current political and economic context in line with the recent publication of the WHO HHGL. Only studies published in English were included.

### Screening and quality appraisal

Search results were screened in Rayyan QCRI, a systematic review screening app. Screening was undertaken by two reviewers (EN, AI) in two stages: 1) title and abstract and 2) full text. Each reviewer screened 10% of the others’ documents and disagreements were resolved through discussion until a consensus was reached. A key discussion was whether studies reported ‘substantive’ data, which was interpreted on a case-by-case basis. Data were substantive if they could be extracted and analysed beyond a single sentence.

Included empirical studies were appraised using the Mixed-Method Appraisal Tool (MMAT) for quantitative, qualitative, and mixed-methods studies (Hong *et al*. 2019). For case studies based on expert opinion or experience, we used an adapted version of the *JBI Critical Appraisal Checklist for narrative, expert opinion and text* (McArthur *et al*. 2015). Both tools use a checklist of questions to aid users to assess study quality. The MMAT tool considers the methodological quality criteria for different study types and the JBI Critical Appraisal Checklist considers the source of text, expert standing, and quality of argument. The checklist was completed by a single reviewer for each study that meet the inclusion criteria (EN).

### Data extraction and synthesis

Summary data for each included study were extracted and tabulated in an Excel sheet. These data included contextual information on the publication (e.g. title, date, authors), policy setting (e.g. country or locality), study methods, the policy (title, organisation, aims), date of implementation (if applicable), and housing-related health hazards targeted by the policy. Studies were grouped by methodological type (e.g. case study) and by health-risk(s) for analysis. Full papers were imported to the Atlas.ti, a qualitative data analysis software, for in-depth qualitative analysis.

An iterative process was followed to explore barriers and enablers for policy development, adoption, and implementation. The process began with familiarisation of the studies, through reading each study manuscript. We used a hybrid inductive and deductive coding approach (Fereday and Muir-Cochrane 2006). Deductive coding used an *a priori* codebook, which contained general categories informed by our prior work (World Health Organization 2021) and previous studies on barriers and enablers to health policy development and adoption (Carmichael *et al*. 2012, Weiss *et al*. 2016). Deductive code categories included economic/financial factors, collaboration, knowledge, policy perception, and policy process and resources. Deductive codes also included policy stage codes that we adapted from the framework from Howlett et al.: agenda setting, formulation, decision-making, and implementation (Howlett *et al*. 2016). However, we adapted to the context of housing, where decision making was also understood as adoption or uptake of policies at different scales (e.g. within a household or by a landlord) rather than just as the decision-making process to establish policy by government or authorities. The *a priori* codebook and definition of terms can be seen in the supplementary material. The inductive coding involved identification of ‘an important moment’ in the data and ‘encoding it prior to interpretation’ (Fereday and Muir-Cochrane 2006). Relevant data were coded with a descriptor of the barriers and enablers (e.g. collaboration), as well as policy stage (e.g. agenda setting) to explore the dynamics of barriers and enablers within the policy process. The coded text was then grouped and explored to generate initial themes. These themes were refined through iterative discussions between those involved in the coding. The thematic synthesis was completed by two individuals, one researcher (EN) led analysis, developed the codebook, and coded all included studies, and a second researcher (KZ) was involved to discuss clarity and meaning of generated codes and themes.

## Results

**Figure 1.**
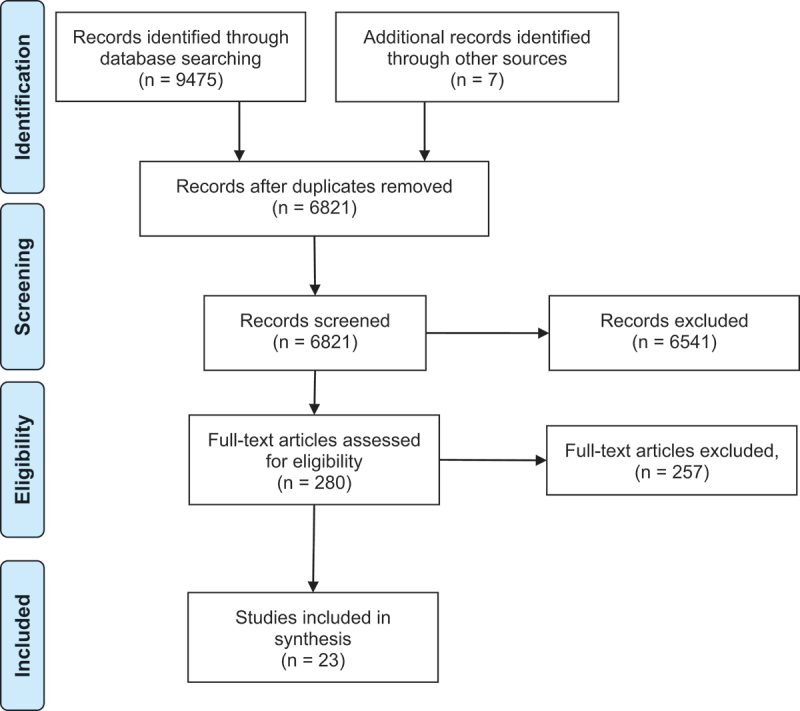
The flow of records in the review.

The flow of records through eligibility screening is shown in Figure 1. 9481 records were identified from searches in bibliographic databases and other sources. Of the 6820 unique records (after duplicates were removed), 279 studies were eligible for inclusion following the appraisal of titles and abstracts. A total of 257 papers were then excluded at the full paper stage on the basis of scope, policy, language, availability, or not reporting substantive data, resulting in 23 studies eligible for inclusion in the thematic analysis.

### Description of included studies

The included studies are summarised in Table 2. All studies were from high-income countries, with the majority from the USA (18), two from Australia and one each from Canada and New Zealand. Over half of papers (13) focused on smoke-free housing policies, three on lead laws, two on housing inspections for health, two on housing quality codes, and one each on accessible housing guidelines and radon control programmes. Ten studies were case studies, often reporting experience of the policy process, six studies were qualitative using focus groups or interviews. The remaining studies were mixed methods (3) or quantitative (3), generally using surveys for data collection. Policy approaches included mandates that aim to effect changes in occupant behaviours through banning smoking indoors, inspection tools, measures of housing quality, and lead removal programmes – including a specialist court to deal with deficiencies and housing codes and design guidelines.

**Table 2.** Included study characteristics.

#	Author(s) and year	Country (and setting)	Policy investigated	Paper focus	Study design and data collection
1	Baezconde-Garbanati et al. (2011)	USA, California	Smoke-free housing policy	Barriers to smoke-free policy in Multi-Unit Homes	Mixed-method: Focus groups; surveys
2	Bain et al. (2016)	USA, Iowa	Radon testing programme	Successes and Challenges in Implementation of radon control activities	Case study
3	Bennett et al. (2016)	New Zealand	Housing quality assessment: rental warrant of fitness	Testing of policy tools	Qualitative: Field test and interviews
4	Burdette et al. (2014)	USA, South Dakota	Smoke-free housing policy	Reasons for smoke-free policy adoption	Quantitative: survey
5	Campbell et al. (2013)	USA, Philadelphia	Lead court	Effectiveness of the Lead Court	Qualitative: Interviews
6	Coyle III et al. (2016)	USA	Housing codes for health	Facilitators and barriers to code amendments proposals	Quantitative: Analysis of historical data
7	Drach et al. (2010)	USA, Oregon	Smoke-free policy	Acceptability smoke-free policies	Mixed methods: Survey and interview
8	Farley et al. (2015)	USA, New York	Smoke-free policy	Correlates and interest in smoke-free policies	Quantitative: Survey
9	Hernandez et al. (2019)	USA, New York	Smoke-free policy	Barriers and perceptions of smoke-free policy	Mixed-methods: Survey, interview, focus group, observational field work
10	Jiang et al. (2018)	USA, New York	Smoke-free policy	Barriers and perceptions of smoke-free policy	Qualitative: Focus groups
11	Jacobs et al. (2007)	USA	Indoor air pollution	Successes and Challenges in implementation of indoor air pollution policy	Case study
12	Kaufman et al. (2018)	Canada	Smoke-free policy	Compliance and enforcement of smoke-free policy	Qualitative: Focus groups and interviews
13	Kegler et al (2019).	USA, Georgia and North Carolina	Smoke-free policy	Implementation and enforcement of smoke-free policies	Qualitative: Interviews
14	Kegler et al. (2018)	USA, Georgia and North Carolina	Smoke-free policy	Development of smoke-free policy	Qualitative: Interviews
15	Korfmacher & Hanley (2013)	USA	Lead policy programmes	Effectiveness of the local lead laws	Comparative case studies
16	Lea & Torzillo (2016)	Australia	Housing inspections for health	Use of data in policy development	Case study
17	Lieb (2018)	USA, Baltimore	Housing codes for housing quality	History of housing-code enforcement	Case study
18	Magavern and Magavern (2018)	USA, Rochester & Buffalo	Lead policy	Effectiveness of lead programmes	Comparative case studies
19	Pizacini et al. (2011)	USA	Smoke-free policy	Programmes for the adoption of smoke-free policy	Case study
20	Ruhe (2019)	USA	Smoke-free policy	Challenges and success in implementing smoke-free policy	Comparative case studies
21	Satterlund et al. (2013)	USA	Smoke-free policy	Barriers to smoke free policy	Comparative case studies
22	Stein et al. (2016)	USA	Smoke-free policy	Issues with smoke-free policy enforcement	Case study
23	Ward & Bringolf (2018)	Australia	Accessibility design guidelines	History of developing guidelines	Case study

### Thematic synthesis

**Figure 2.**
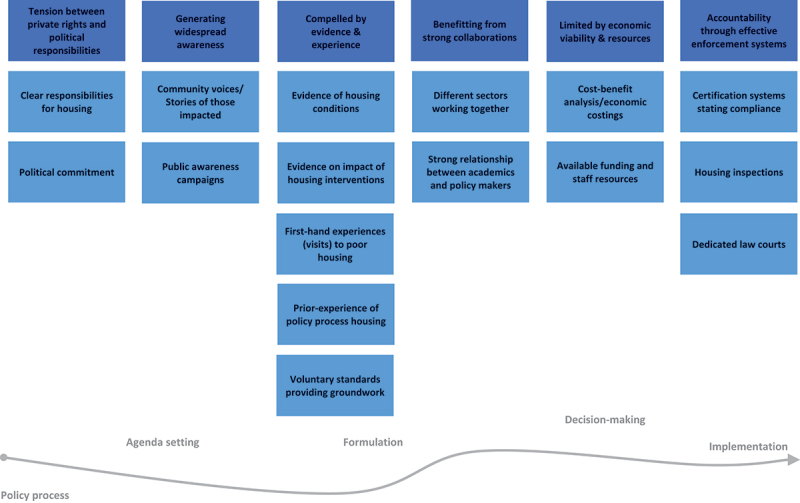
Themes and specific enablers plotted at their relative position along the policy process.

In this section, we describe the results of the thematic synthesis which produced six themes, summarised in Table 3 with their relationship to the policy stage. Each theme is described as a sub-section of the results, drawing on illustrative examples from the included studies. An overview of the themes is plotted along the policy process and specific factors that enable policy development and implementation are illustrated in Figure 2. While most themes spanned all policy stages (Table 2), the theme related to economic viability and resources was linked to decision making and implementation stages and accountability through effective enforcement systems only linked to implementation.

**Table 3.** Relationship between policy stage and themes.

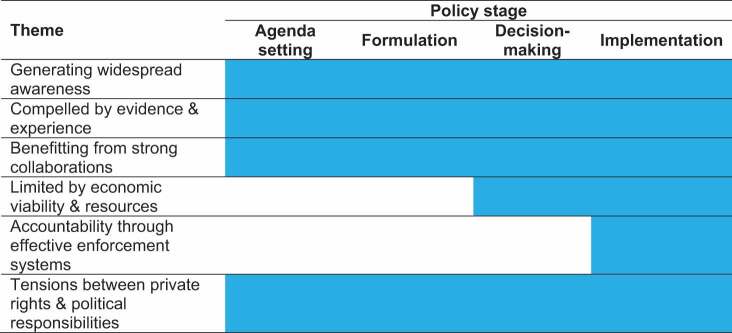

### Tensions between private rights and political responsibilities

As in other public health policy areas (e.g. seatbelts and tobacco), the included studies highlighted a tension in healthy housing policy between individuals’ private rights and interests and political responsibilities. Arguments related to personal rights and liberties (Drach *et al*. 2010) unclear responsibilities (Jacobs *et al*. 2007) and market forces driving housing design and maintenance (Lieb 2018, Ward and Bringolf 2018) constrained the development and implementation of healthy housing policy, which are underlined by a lack of political commitment by national and local governments to housing.

Included studies highlighted how unclear responsibilities for housing, and where responsibilities were left to market dynamics, resulted in the lack of enactment of broad standards across all housing. For example, in the United States, Jacobs et al. (2007) discussed how low public demand for standards was due to diffuse responsibilities for the health impacts of housing between actors (architects, engineers, surveyors, developers, property managers, homeowners, tenants, and others), which was in contrast to other environmental health risks, where the ‘Polluter Pays’ principle holds the originating polluter accountable (Jacobs *et al*. 2007). These unclear responsibilities typically led to owners and tenants turn to courts for specific individual harms, rather than to campaign through political processes to demand standards that cover all housing (Jacobs *et al*. 2007). In Australia, individual home buyers were unlikely to pay for additional costs for accessible design features (e.g. handrails) for the ‘common good’, particularly where there was no immediate benefit for them and thus setting the agenda for policy was seen as particularly challenging (Ward and Bringolf 2018). Thus, Ward & Bringolf (2018) argue how relying on market dynamics to drive compliance with voluntary healthy housing guidance hindered the adoption of accessible housing: *‘relying solely on demand, particularly in the housing market, is problematic when addressing issues of inclusion, participation and rights’* (Ward and Bringolf 2018). Similarly, private housing market dynamics in Baltimore, USA, were said to led to poor housing conditions and allowed *‘sellers to evade responsibility for substandard conditions’*, leaving the burden on people who could not afford remediation measures (Lieb 2018). These tensions between government responsibilities, public demand, and market dynamics mean that in many countries there been an inconsistent inclusion of health aspects on the housing policy agenda.

Issues around individuals’ rights of residents and authorities’ responsibilities (both landlords and government) impeding on personal freedoms were commonly raised in included studies discussing smoke-free housing policies. For policies prohibiting smoking at home, these issues manifested in multiple ways across different actors, from tenants challenging: *‘This is my home. You can’t tell me what to do in my home!’* (Drach *et al*. 2010); to landlords questioning their prerogative to invade personal freedoms (Satterlund *et al*. 2013, Burdette *et al*. 2014); and to neighbours feeling uncomfortable telling people what they cannot do (Baezconde-Garbanati *et al*. 2011) with resistance strongest from those who smoke (Baezconde-Garbanati *et al*. 2011, Satterlund *et al*. 2013, Farley *et al*. 2015, Jiang *et al*. 2018, Hernández *et al*. 2019). Landlords further raised concerns about government intrusion into people’s homes and not making the wider use of tobacco illegal instead (Satterlund *et al*. 2013). Additionally, fears about the unequal impact of such policies on specific rights of low-income and smoking populations were a barrier to uptake (Satterlund *et al*. 2013, Campbell *et al*. 2013, Jiang *et al*. 2018). These tensions between individual rights and freedoms meant the uptake of smoke-free housing policy was often resisted and the implementation was challenging.

Studies highlighted that policy uptake and implementation was enabled where rights and responsibilities were clearly defined. For example, the Municipal Code of Chicago, USA, which grants health officials the authority to access housing to inspect lead hazards was seen to positively enable protection against exposure to lead (Korfmacher *et al*. 2013). However, access could be impeded by residents’ rights and the need to give 24 hours-notice, which then prohibited the gathering of evidence required for enforcement, particularly in the case of smoke-free policies (Berg *et al*. 2016). Korfmacher & Hanley (2013) found that where residents had the right to request inspection or action from landlords, this enabled policy uptake and implementation, as this gave power to the resident to hold authorities responsible to deal with lead hazard (e.g. remove lead hazards) (orfmacher *et al*. 2012).

### Accountability through effective enforcement systems

Studies reported that healthy housing policy needs to sit within effective enforcement systems that create a culture of accountability for successful implementation. A lack of effective enforcement mechanisms was seen as a challenge to policy implementation (Satterlund *et al*. 2013, Jiang *et al*. 2018, Hernández *et al*. 2019). Challenges reported in the studies included a lack of responsiveness from housing owners and authorities in dealing with violations to smoke-free policy (Jiang *et al*. 2018, Kaufman *et al*. 2018, Hernández *et al*. 2019), which led to undermining the system, poor administration – leading to a reduction in budgets (Lea and Torzillo 2016) and lengthy grant applications (Campbell *et al*. 2013) and processes (Campbell *et al*. 2013) resulting in delays to compliance and risking continued exposure to harms. Studies found that policy scope was a barrier to enforcement, for example: the stipulated thresholds for lead hazards restricted enforcement, although health risks likely remained with inaction (Magavern and Magavern 2018); a lack of legal housing titles made it impossible to access grants (Campbell *et al*. 2013) or persons at risk (such as those with elevated lead blood levels) were not listed on rental leases and so were not covered through established procresses (Campbell *et al*. 2013). Other barriers included institutions without adequate resources to handle cases (Campbell *et al*. 2013) and over flexibility of the system creating ineffective action (Korfmacher *et al*. 2013).

Systems tailored to support policy enforcement were found to enable effective implementation. Examples include Philadelphia’s Lead Court (PLC), a specialised court dedicated to deal with lead removal orders, which provided dedicated judges and specialised legal tools, such as checklists for the court procedures (Campbell *et al*. 2013). The PLC was found to be more effective than pre-court enforcement strategies (Campbell *et al*. 2013) reasons for this success included the use of clear forms enabling court processes, use of fines as incentives, judges’ knowledge of harm from lead in homes, funding to support remediation work, speed of processing and law officials and health departments working together (Campbell *et al*. 2013):

Studies found that the use of penalties and tools, such as certification systems, supported the effective enforcement of policy. Penalties that kept landlords accountable to local lead laws in the USA, included fines for violations (Campbell *et al*. 2013, Korfmacher *et al*. 2013) tenants having the ability to withholding rent until work is completed (Campbell *et al*. 2013) and the possibility of landlords being sued (Korfmacher *et al*. 2013). Fines also encouraged tenant compliance to smoke-free policies across the USA (Kegler *et al*. 2018) as did the loss of deposit money from tenants (Burdette *et al*. 2014) and evictions for repeat offenders of no-smoking policies (Hernández *et al*. 2019). Tools found to support compliance included certificates that confirmed the house was safe from hazards (i.e. lead free), and in particular, integrating these into requirements needed for rental and insurance (Korfmacher *et al*. 2013) the use of verbal (Stein *et al*. 2016) and written (Kaufman *et al*. 2018) warnings from landlords to tenants and including no-smoking clauses within contractual agreements (Baezconde-Garbanati *et al*. 2011). Clear forms that were easy to fill out were seen as beneficial (Bennett *et al*. 2016), as were the specific printed menus that helped to speed up cases (Campbell *et al*. 2013).

Housing inspections were reported to enable compliance to policy in the included studies. For example, inspections were viewed as simple but very effective in supporting the implementation of lead laws in the USA (Korfmacher *et al*. 2013):

This simple element, readily duplicable in other jurisdictions, fills a basic gap in the primary prevention mission. While many communities conduct ongoing awareness campaigns on lead paint hazards, they have limited primary prevention potential unless tenants have the ability to get their homes inspected.

Housing inspections were used to identify lead hazards, these were often proactive in high-risk areas (Magavern and Magavern 2018), as well as part of routine checks for insurance certificates (Korfmacher *et al*. 2013). Similarly, inspections were employed to detect smoking violations, often as part of maintenance and housekeeping visits (Stein *et al*. 2016, Kegler *et al*. 2019). Where tenants received compensation for costs related to inspections and remediation work, as well as harm, damages, and court fees were reported to increase the reporting of violations (Korfmacher *et al*. 2013).

Phased implementation was also found to aid implementation, by giving prior warning before the policy went into effect (Pizacani *et al*. 2011, Kegler *et al*. 2019). Linking to existing rental and legal systems was found to be beneficial. Linking to rental processes was reported as an enabler to compliance of lead policy in Buffalo, USA, where lead free certification is required when housing owners register as part of the Rental Registration Law and inspections can be required before moving under residential assistance programmes (Magavern and Magavern 2018). Although changes to rental contacts after tenancy was established were deemed to negatively impact implementation (Drach *et al*. 2010). Similarly, linking federal lead requirements within existing local laws increased effectiveness (Korfmacher *et al*. 2013).

Training and technical assistance for stakeholders involved in policy implementation were reported as an enabler in the included studies. This included training of assessors to inspect housing for hazards (Bennett *et al*. 2016) education for housing owners, employees and tenants on the lead harms (Magavern and Magavern 2018) and providing education and technical assistance about each step of the implementation process for smoke-free policies (Ruhe 2019). Furthermore, additional support for smokers to quit was deemed helpful for the success of smoke-free policy (Jiang *et al*. 2018, Kegler *et al*. 2019, Ruhe 2019).

### Limited by economic viability & resources

Studies underlined that the success of policy processes was linked to economic feasibility and availability of resources. For example, in the USA, high costs to obtain certificates were found to prohibit compliance to lead laws (Korfmacher *et al*. 2013), and a lack of finance restricted the ability to realise housing repairs (Lieb 2018) and to employ staff to support enforcement (Hernández *et al*. 2019). Where sufficient funding was provided to support policy programmes, this was viewed as vital to its success (Bain *et al*. 2016, Magavern and Magavern 2018). This included funding to local authorities to operate policy programmes on radon (Magavern and Magavern 2018), as well as specific funding for meetings, workshops, and education materials (Pizacani *et al*. 2011) or to support housing owners ordered to make repairs (Campbell *et al*. 2013).

Economic concerns of policy impacts on profits led to resistance to uptake of smoke-free policy from housing managers and housing owners (Satterlund *et al*. 2013) and accessible design guidance by the building industry (Ward and Bringolf 2018). The expected impact of the policy on rental income affected policy uptake, with landlords citing concerns of reduced rental income (Baezconde-Garbanati *et al*. 2011, Satterlund *et al*. 2013, Burdette *et al*. 2014, Stein *et al*. 2016); as homeowners were ‘worried about [impacts to] the bottom line’ (Satterlund *et al*. 2013) from increased vacancies. However, reduced maintenance (Pizacani *et al*. 2011) and turnover (Farley *et al*. 2015) costs related to smoking tenants were seen as beneficial, enabling uptake of smoke-free policies:

We learned that continually making the business case for smoke-free housing was an effective motivational strategy for moving stakeholders through the early stages of change (precontemplation, contemplation, and preparation) into making and maintaining the change. (action and maintenance)

Builders were reported to charge higher prices for accessible housing, which deterred buyers requesting accessible features (Ward and Bringolf 2018). As a result, universal design was uncommon in mainstream housing, as businesses, suppliers, and institutions were focused on *‘the short-term outcome of maximized profit at the point-of-sale’* (Ward and Bringolf 2018)

Studies found that staff resourcing were challenges in the adoption and implementation of policy, this included; landlords citing increased workloads of housing staff as reasons for not adopting smoke-free policy (Satterlund *et al*. 2013, Burdette *et al*. 2014). Similarly, Campbell et al. (2013) found considerable staff time and resources for inspections, and paperwork was a challenge in the implementation of lead laws.

### Compelled by evidence and experience

Studies found that evidence and experience of healthy housing and of policy processes successfully supported policy development and implementation. Multiple types of evidence and data were employed through multiple pathways, this included both evidence on health impacts of housing (Korfmacher *et al*. 2013, Farley *et al*. 2015, Magavern and Magavern 2018) and evidence of current housing conditions (Lea and Torzillo 2016, Lieb 2018). Evidence from scientific research, in particular, was reported to meaningfully support policy formulation (Lea and Torzillo 2016).

Decision makers’ knowledge and awareness of health benefits were found to be a motivator to policy uptake (Drach *et al*. 2010, Campbell *et al*. 2013, Farley *et al*. 2015, Kegler *et al*. 2018) this included landlord knowledge on the benefits to the safety of staff involved in the management of buildings (Pizacani *et al*. 2011) as well as knowledge of co-benefits such as the reduced risk of fire from smoke-free policy (Kegler *et al*. 2018). Evidence showing residents support for smoke-free housing helped motivate housing owners and managers to adopt policy (Pizacani *et al*. 2011). Evidence that highlight gaps in national policy on accessible housing in Australia helped set the agenda and stimulated action for the development of accessible housing guidelines (Ward and Bringolf 2018). Where evidence was used to define standards for health, this was found to help set the policy agenda in Australia (Lea and Torzillo 2016) aid smoke-free policy formulation in USA (Kegler *et al*. 2018), as well as help motivate the uptake and support implementation for inspections, as standards provided a benchmark to compare against (Magavern and Magavern 2018). Similarly, international standards and external targets on disability rights’ supported the setting of policy agenda for accessible housing in Austrialia (Ward and Bringolf 2018). In Australia, voluntary standards on accessible design were later adopted in official regulations (Ward and Bringolf 2018). A lack of evidence was found to hinder the implementation of smoke-free housing policy as it was difficult to verify violations and thus enforce the policy (Hernández *et al*. 2019, Kegler *et al*. 2019). Evidence was also found to be key in helping to decide where to apply programmes. For example, New York State, USA employed health data on elevated blood lead levels to declare high-risk neighbourhoods, following which inspections and repair orders were issued (Korfmacher *et al*. 2013, Magavern and Magavern 2018).

Studies found that experiences of housing conditions and policy processes elsewhere supported policy development and implementation. For example, first-hand experience of current housing conditions in Baltimore, USA, was found to motivate government officials to push through policy: *‘What I have seen appalls me’* (Lieb 2018). Drawing on others experience with smoke-free housing policy elsewhere was found helped to support policy formulation, this included gathering insights of others’ policies as well as liaising directly with members in housing associations on their experiences (Kegler *et al*. 2018). Previous experience in policy development was found to be correlated with acceptance of proposals for changes to US building codes (Coyle *et al*. 2016), highlighting how prior experience of the system led to successful changes. Advice from others with experience and health experts was sought regarding the development and implementation of policy. Experience of implementing policy was found to be helpful in the decision-making stage for housing managers considering smoke-free policy (Satterlund *et al*. 2013). Advice from health departments was sought in the formulation of policy, particularly around survey design, policy language, as well as presentations on the rationale for policy (Kegler *et al*. 2018).

### Generating widespread awareness

Creating awareness for the topic of health and housing as well as awareness of policy existence was reported in the literature as an enabler to development and implementation.

Individuals and organisations advocating for improved health and housing policy were widely reported as an instigator to policy development. This included individuals in positions of authority, such as mayors, helping to set the agenda needed for policy formulation (Lea and Torzillo 2016, Lieb 2018, Ward and Bringolf 2018). There are cases were community action was vital in setting the agenda for housing policy (Korfmacher *et al*. 2013, Ward and Bringolf 2018): *‘The law was the culmination of a grassroots community effort formed in response to community concerns’* (Korfmacher *et al*. 2013). This often-involved groups adversely affected by harms:

… those who take political responsibility; they take public and collective action to intervene, and call to account those people who could do something, but who do nothing. Typically, they are led by those who are most affected, who understand personally what social injustice means (Ward and Bringolf 2018).

Advocacy groups for people living with disabilities played a prominent role in Australia to develop first voluntary guidelines for accessible design, which were later established in regulatory codes (Ward and Bringolf 2018). In Rochester having a non-profit organisation lead advocacy was found to be a benefit in establishing local lead laws (Magavern and Magavern 2018). However, the relatively low priority of the issues of housing and health within the local context was reported to be a barrier to uptake and implementation, as it was deemed insignificant compared to other issues (Jiang *et al*. 2018, Lieb 2018).

Public demand was widely found to be a factor influencing policy uptake (Pizacani *et al*. 2011, Korfmacher *et al*. 2012, Farley *et al*. 2015, Coyle *et al*. 2016, Kegler *et al*. 2018, Ward and Bringolf 2018). Publicity campaigns were used to generate awareness of housing hazards and policy programmes. In Iowa, USA, messages about radon harms via brochures, social media, videos, and news stories on television and radio helped to increase the uptake of radon testing and mitigation (Bain *et al*. 2016). Education about the laws on lead paint and the availability of grants was similarly reported to be effective in raising awareness about processes in the Philadelphia Lead Court (Campbell *et al*. 2013). Newsletters, flyers, meetings, and resident orientations used to announce and promote smoke-free housing policy leading to smoother implementation (Jiang *et al*. 2018, Kegler et al. 2018, 2019). However, messaging used needed to be favourable to create positive responses (Pizacani *et al*. 2011) and often had more success if it was through trusted communication channels (Pizacani *et al*. 2011, Kaufman *et al*. 2018).

Awareness of legal backing of smoke-free policies was found to be correlated with policy uptake (Farley *et al*. 2015). On the other hand, a lack of awareness of a policy and its associated services was viewed as a barrier to uptake and implementation (Kaufman *et al*. 2018). Lack of awareness of provisions in the housing code was said to be a paralyzing problem, as judges did not realise, they were able to apply jurisdiction over violations, which then failed to keep landlords and housing owners to account:

‘I had no idea there were such provisions in the housing code’, said one property owner to a judge. ‘To tell you the truth’, the judge replied, ‘neither did I’ (Lieb 2018)

### Benefitting from strong collaborations

Collaboration between multiple actors was found to have enabled policy development and implementation, particularly cross-sectoral partnerships between health and housing actors. For example, health departments played a crucial role in the implementation the U.S. Department of Housing and Urban Development smoke-free housing rule, by acting as a ‘neutral convener’ with public housing associations (Ruhe 2019). Cross-sectoral collaborations between health and legal actors were also viewed as being effective, for example, PLC ‘‘provide[d] effective interdisciplinary use of health and law together” to deal with lead violations (Campbell *et al*. 2013).

Where community engagement occurred in policy processes this was found to help strengthen development and implementation. It was particularly highlighted during the policy formulation stage, where residents provided feedback on the policy design, and during implementation when residents took a role in reporting violations (Stein *et al*. 2016, Jiang *et al*. 2018, Kegler *et al*. 2018, Ruhe 2019). Collaborations between institutions were also found to help focus policy efforts, this included increasing the reach of policy information campaigns (Bain *et al*. 2016) helping scale-up policy to further locations (Lea and Torzillo 2016) and supporting the development of criteria for housing quality to cover multi-sectoral domains (Telfar-Barnard *et al*. 2017).

The nature and timing of collaboration was reported as important. Collaboration at the onset was viewed as most effective to support landlords ‘through the stages of change into the implementation of smoke-free policies’ (Pizacani *et al*. 2011). Cooperation that was constructive was also viewed as positive:

To another respondent, “That type of cooperation — … trying not to penalize people but trying to help people — [is] probably one of the best things I’ve seen in the city and in the court systems”. (Campbell *et al*. 2013)

Investing time in developing a relationship with key decision-makers, such as housing managers and landlords, was widely found to support decision-making for the uptake of smoke-free policy (Pizacani *et al*. 2011, Satterlund *et al*. 2013, Ruhe 2019). This including building trust and willingness to work through difficult issues with housing managers in the adoption of smoke-free policy (Pizacani *et al*. 2011). A lack of access to decision-makers was seen to prohibit policy uptake, resulting in the need to adjust outreach strategies (Satterlund *et al*. 2013). Similarly, staff turnovers affected policy development as relationships were lost with the change in staff member (Satterlund *et al*. 2013).

Where policy could negatively impact relationships, this was viewed as problematic and likely to affect uptake and implementation of the policy. This included relationships between tenants and housing owners (Baezconde-Garbanati *et al*. 2011, Satterlund *et al*. 2013, Burdette *et al*. 2014, Jiang *et al*. 2018) as well as relationships between neighbours (Baezconde-Garbanati *et al*. 2011, Jiang *et al*. 2018, Kaufman *et al*. 2018) around the uptake and enforcement of smoke-free policy, and led to resistance of smoke-free policy from tenants (Farley *et al*. 2015).

## Discussion and recommendations

Given renewed calls for address substandard housing and to update housing standards, along with gaps in healthy housing policy globally, we aimed to understand what enables the development and implementation of healthy housing policy to generate lessons that can support implementation of the recently published WHO HHGL and development of healthy housing policy in locations where it is currently lacking. Our study examined the development and implementation of healthy housing policy, through a review and thematic synthesis of available evidence. We found that healthy housing policy processes are complex, and while policy processes varied across geographical locations and housing-related health risks, there were common barriers and enablers to policy development and implementation. Key findings were as follows.

Firstly, we found that raising awareness of housing and health issues enabled both the development and implementation policy. Community and grassroots organisations played a crucial role in setting policy agendas through campaigning on housing-related health risks. Where local government raised awareness of policy existence this increased policy uptake. Raising awareness of housing-related health risks will therefore likely increase public demand for healthy housing, which in turn will increase support for policy development and implementation. Secondly, and linked to awareness, our findings revealed how evidence on health impacts of housing and experience of housing conditions helped set the agenda and compelled healthy housing policy uptake, and experience in policy processes helped ease adoption and implementation. We argue therefore for increased evidence on the health impacts and co-benefits of improved housing, which can steer discussions with decision makers to encourage the development and implementation of healthy housing policy. Similarly, Carmichael (2020) highlighted the lack of health evidence in building regulations and recommended creating awareness of public health evidence and using a systems approach to improve standards (Carmichael *et al*. 2020). Pineo et al. (2020) described how evidence of the health impacts of the built environment (including housing risks) were used in planning policy development and implementation in the USA and Australia, specifically to negotiate better design outcomes with housing developers (Pineo *et al*. 2020). Further research could explore which types and formats of evidence, over which timescales, are useful for housing policymakers in specific contexts (e.g. knowledge translation products like evidence briefs).

We found that collaborations involving multiple and diverse actors strengthened policy development and implementation. Cross-sectoral relationships between housing and health professionals enabled interdisciplinary knowledge sharing and collaboration between communities and decision makers (landlords and governing authorities) which helped address difficult issues in implementation. The importance of collaboration is supported by other studies of healthy urban development, such as Pineo and Moore (2021) who found that relationships between public officials, developers and design times were particularly important in achieving health-promoting design outcomes in settings where building codes were lacking such requirements (Pineo and Moore 2021). Investing time to develop relationships with key actors should be central in healthy housing policy development and implementation and involvement of communities impacted by policy will also likely help to ensure inclusivity and policy effectiveness.

Economic concerns were repeatedly raised as a barrier to policy adoption and implementation, including both impacts on profits (e.g. rental income) and due to lack of available resources to implement policies. Similarly, the dominance of private housing markets has resulted in a lack of investment in housing adaptions to meet long-term population needs. For example, demographic change calls for refurbishments to ensure people can continue to live independently at home. However, embedding ageing considerations in housing has been difficult due to dependency on individual financing. Evidence that demonstrates long-term economic viability or that counters common concerns around reduced rental income would help strengthen arguments for policy adoption. Specific financial assistance would strengthen the implementation of policy, particularly were increased staff resources are required or where there are long-term benefits beyond the current occupier (such as for accessible design). Parallels can be drawn with zero-emission housing (ZEH) innovations, which suffer similar financial constraints and would be supportive of health. Moore et al.’s review of ZEH policy innovations in the EU, UK, and California showed financial support through ‘measures to reduce capital costs through economic efficiencies in the material and building process and through the introduction of financial mechanisms such as low interest loans or tax breaks (reduced stamp duty for example)’ (Moore *et al*. 2014).

We found that successful implementation of policy was underpinned by the effectiveness of enforcement systems. Effective mechanisms that supported policy implementation included housing inspection used to identify harms, the use of tools (e.g. certificates documenting compliance) and incentives (e.g. fines). Where policies were aligned with legal systems, for example courts or rental agreements, or health authorities, such as through flagging exposure to harm, this was found to particularly support enforcement. Therefore, connections between healthy housing policies and legal systems should be critically appraised and draw on the successful tools and incentives used elsewhere for effective implementation of policies

Finally, our findings uncovered tensions between private and public rights and political responsibilities. Housing was often seen as an individual right not to be interfered with, with responsibilities often left to private housing markets due a lack of political commitment from national and local governments. This in turn has led to social context that housing is not something to be provided or regulated by the state and has resulted in limited public demand for policy that covers healthy housing design and maintenance. The included papers represent four countries and may not represent the dynamics in countries where public housing provision is more predominant. However, Werna et al. (2022) similarly noted tensions between private housing developers and regulations, with housing developers often able to pay to avoid regulations resulting in non-compliance (Werna *et al*. 2022). While housing development and maintenance remains strongly influenced by private markets, outside of public policy, the development and implementation of healthy housing policy will likely remain challenging. Addressing this tension and recognising healthy housing policy as a universal public requirement will likely result in the increased development, uptake, and more effective implementation of housing policy that benefits all. This requires political commitment of national and local governments, without which the right to adequate housing will not be fully realised (Nikuze et al. 2019; United Nations 2017).

Our findings overlap with research on the facilitators and barriers to policy development and implementation in other policy areas, and in essence, inserting health considerations into housing policy encounters similar difficulties, such as the need for appropriate resources and collaboration among stakeholders. For example, facilitators and barriers to health-promoting policies included: collaborative decision-making, effective leadership, availability of resources and trained and knowledgeable staff (Weiss *et al*. 2016). Similarly, Carmichael *et al*. (2012) grouped barriers and facilitators to incorporating health and wellbeing in spatial planning by four factors: knowledge, partnership, management and resources and policy process (Carmichael *et al*. 2012). While this reinforces our finding, learnings from policy development and implementation across other sectors and locations will also likely help guide healthy housing policy as well, particularly if there is knowledge relevant for the local context. Several guidance documents from health and built environment organisations address these challenges and make recommendations for policy development and implementation. The Canadian Institute of Planners & HP Lanarc-Golders, (n.d.) guidance calls for collaboration and integrated policy-making to ensure built environment components are not considered in isolation (Canadian Institute of Planner). A Royal Institution of Chartered Surveyors report (Pineo and Rydin 2018) recommends demonstrating that costs and benefits of healthy housing are paid by different actors from those who reap the benefits (financial and otherwise) to counter concerns that added costs will not be adequately covered by future returns (through sales or leasing of new homes) (Pineo and Rydin 2018).

Our study was limited by a lack of diversity in available literature. All included studies were from high-income settings and represent four countries, with the majority from the USA (19/23), two from Australia and one from New Zealand and Canada. While we did not directly limit to non-English speaking countries, our inclusion criteria to select studies in English may have indirectly created bias towards English speaking countries. Similarly, as we predominately used terms that were referenced on the WHO HHGL as search terms, this may have not been encompassing of language and terms used elsewhere – which may have led to further indirect bias. Over half of the included studies were focused on smoke-free housing policy that focus on prohibiting residents’ behaviour, these policies likely experience difference challenges compared to policy which stipulates building standards. As we limited our review from 2010 onwards, to ensure findings were applicable currently, we will not have captured historical lessons prior to 2010. Expanding this review and targeted research on additional health-risks and in middle and low-income countries may enhance understanding for additional contexts, however, themes identified may provide learnings for policymakers, researchers, and programme officers working on healthy housing policy. Furthermore, we did not consider the effective of policies for health, which was deemed outside the remit of the review, we are therefore not able to conclude whether effective policy implementation results in health benefits.

We cannot establish if these findings will be transferable to different countries, particularly low-income settings where political and socio-economic conditions and thus housing dynamics vary dramatically. However, for countries where policies are lacking, such as in LMICs, establishing an agenda on issues of health and housing is the first step to develop policy. Our findings suggest that generating awareness, evidence and first-hand experience at this intersection and working in cross-sectoral partnerships enabled agenda setting. Transdisciplinary research and programmes that link experts with policy makers and households to generate evidence on housing conditions and prevalence of health hazards may help set this agenda. In particular, stories of lived experience of poor housing conditions may provide powerful motivation for action. Indeed, transdisciplinary approaches are argued as appropriate to shift persistent urban health challenges by bringing policy and practice together for concerted action (Lawrence 2022). Programmes could use similar transdisciplinary approaches as developed by Nix et al (2019), that adapted housing health risk assessments from developed settings and combined with participatory methods to evaluate and design housing with community members and local policymakers and practitioners (Nix *et al*. 2019). Additionally, raising the issue of housing and health on the international agenda could be enabled by linking to existing related platforms, such as international platforms for climate action. Evidence of polices elsewhere, such as examples provided in the WHO review (World Health Organization 2021) and modelling of health and economic impacts of housing, may then enable policy formulation and support decision-making towards adoption. Additionally, action taken individuals and/or developers to implement voluntary standards, such as WELL – which was designed to promote health and well-being in buildings, may provide the ground work and experience for future policy uptake in countries. While many developing nations lack the ‘management and regulatory and monitoring capacities’ (Bredenoord and van Lindert 2010) to ensure policy enforcement, tools such as certification and inspections programmes, could support enforcement. These tools and programmes could be developed in partnership with existing community programmes to help create a culture of accountability and act as form of local advice, examples of this include community health workers to track and provide advice on access clean fuels to reduce household air pollution (WHO 2023).

## Conclusions

The WHO housing and health guidelines highlight the health impacts of housing and provide evidence-based recommendations to promote health through housing. Housing policies that stipulate requirements for health are essential in reducing the disease burden associated with poor housing conditions. Public health and housing advocates have renewed calls to address substandard housing conditions and highlight the need to update standards. At the same time, there is significant lack of healthy housing policy and research globally, particularly in low and middle-income countries. Further to this, due to migration and population growth, it is predicted that 3 billion people will live in informal settlements by 2030 and will be in dire need of access to affordable and healthy housing. Given these gaps and the global urgency for healthy housing, we completed a focused systematic review and thematic synthesis of the barriers and enablers to housing and health policy. Findings from this review can support its development and implementation of healthy housing policy elsewhere.

Despite limitations in the quantity and quality of the evidence, our findings demonstrate that healthy housing policy was facilitated by awareness on issues of housing and health. This was particularly the case where this resulted in public demand for policy, evidence on current housing conditions and health impacts of interventions, collaborations across health, legal and housing sectors, and between residents and decision-makers (landlords and authorities) and through effective enforcement systems that employ incentives, tools such as certificates for compliance, and housing inspections. Challenges to policy development and implementation included concerns around economic viability and impacts on profits (e.g. rental income) and tensions between housing rights and responsibilities, with governments often relying on the housing market to drive design and the maintenance. Understanding these factors and ensuring they are consistently taken into consideration during the development, adoption and implementation of healthy housing policies will be crucial to improve their effectiveness and uptake. Further understanding of successes of healthy housing policy development and implementation in additional settings, principally in low-and middle-income countries, is vital to spur policy in countries with similar geographical, socio-economic and/or cultural conditions. Context-specific research that works with policy-makers and households, to generate awareness and evidence on housing and health will support agenda setting for policy in additional settings and may provided the first step in addressing the gap in the existence of housing policy. This focused systematic review provides a useful starting point for informing future policy processes aimed at providing healthy and equitable housing for all.

## Supplementary Material

Supplementary material_review.docx

## Note

1. Interpretations of the term ‘policy’ vary internationally and there are a variety of regulations and standards that apply to both the development and management of housing, which may be compulsory, voluntary and incentivised by governments. Public policy is broadly defined as ‘the deliberate decisions – actions and nonactions – of a government or an equivalent authority toward specific objectives’, including examples such as ‘statutes, laws, regulations, executive decisions, and government programs’ (Weible 2017).
