# Supplementary material for: Getting to effective housing policy for health: a thematic synthesis of policy development and implementation
Source: Cities Health. 2024 Apr 4;8(3):486–503. doi: 10.1080/23748834.2024.2328951 (PMC11441398; doi:10.1080/23748834.2024.2328951)
Supplement: Supplementary material_review.docx [file RCAH_A_2328951_SM1529.docx]

**Supplementary material**

1. **Example search strategy**

The following section details an example search for the literature as completed in a selected database.

**Search Strategy for MEDLINE**

Ovid MEDLINE(R) and Epub Ahead of Print, In-Process & Other Non-Indexed Citations and Daily Citations via OVIDSP
Searched on: 11^th^ November 2019
Records: 1,114

| **Set** | **Results** | **Search details** | **Category** |
| --- | --- | --- | --- |
| 1 | 7,453,271 | (health* or illness* or medical condition or mental* or well-being or well being or disease* or condition* or impairment or disability).ab,ti. |  |
| 2 | 73,862 | (housing condition* or living condition* or habita* or adequate housing or housing quality).ab,ti. |  |
| 3 | 13,707 | ((hous* or dwelling* or habitation or home*1 or residen* or apartment* or flat* or bungalow* or homeowner* or rent* or tenan*) adj5 (polic* or code* or act or standard* or guideline* or guidance* or regulation* or legislation*)).ab,ti. |  |
| 4 | 762,029 | (air condition* or temperature* or indoor heat or hot spell* or heat wave* or heatwave* or indoor temperature* or heat or heat exposure or thermal comfort or thermal condition*).ab,ti. |  |
| 5 | 426,700 | (heating system* or central heating* or indoor cold* or cold spell* or winter* or cold exposure* or indoor temperature or cold or cool or warm or insulat* or thermal comfort or thermal condition* or permeability or airtightness or air change rate or leak*).ab,ti. |  |
| 6 | 1,381,316 | (accident* or hazard* or safe* or electric accident or electrocution or explosion or fall* or home accident or structural collapse or accident prevention or "home safety" or inhalation or fire or "fire protection" or hot surface or smoke detector or fire blanket).ab,ti. |  |
| 7 | 1,868,975 | (accessib* or functional* or disab* or "retirement home" or elder* or "care home" or assistive or "universal design").ab,ti. |  |
| 8 | 72,920 | (water quality or water tank or contaminated water or water storage or water collection or water tanker or taps or pipes or drinking water).ab,ti. |  |
| 9 | 544,046 | (air quality or pollut* or smoke or particulate matter or carbon monoxide or ventilation or extract fan or exhaust or smoking or cooking fuel or heating fuel or solid fuel* or biomass fuel or cow dung or wood or chimney or stove hood or combustion or kerosene or second-hand smoke or second hand smoke or coal).ab,ti. |  |
| 10 | 455,271 | (damp or mould* or mold or humid or thermal bridge or insulat* or ventilation or permeability or airtightness or air change rate or leak* or gutter* or drainage).ab,ti. |  |
| 11 | 21,374 | (overcrowd* or crowd* or shared occupation or multiple occupation or shared room).ab,ti. |  |
| 12 | 6,493 | radon.ab,ti. |  |
| 13 | 12,540 | asbestos.ab,ti. |  |
| 14 | 131,205 | noise.ab,ti. |  |
| 15 | 582,792 | lead.ab,ti. |  |
| 16 | 5,242,925 | 4 or 5 or 6 or 7 or 8 or 9 or 10 or 11 or 12 or 13 or 14 or 15 |  |
| 17 | 17,018 | housing/ |  |
| 18 | 2,374 | policy/ |  |
| 19 | 29 | 17 and 18 |  |
| 20 | 13,727 | 19 or 3 |  |
| 21 | 7,498,852 | 1 or 2 |  |
| 22 | 2,586 | 21 and 20 and 16 |  |
| 23 | 1,114 | limit 22 to (humans and yr="2010 -Current") |  |

**Key:**

/ = indexing term

* = truncation

.ti,ab. = terms in either title or abstract fields

adj5 = terms within five words of each other (any order)

* = truncation

1. **Codebook**

This section details the a priori codes used for deductive coding. A definition and reference are provided for each code. Table S1 details the definition of the policy stage and Table S2 details codes describing barriers and enablers to policy development and implementation.

**Table S1: Codes describing the stages on the policy process**

| Policy stage | Definition | Reference |
| --- | --- | --- |
| Agenda-setting | Agenda setting concerns the process by issues are given attention and focuses on the determination and definition of what constitutes the problem that subsequent policy actions are intended to resolve. | Howlett^1^ |
| Policy formulation | The creation of a new policy to support discovery of which solutions are most suited to addressing the problem at hand |  |
| Decision-making | Decision making involves the selection of a course of action from a range of policy options |  |
| Adoption/uptake | Adoption of policy by at different scales: landlord, build new buildings- |  |
| Implementation | Putting a policy into practice: clear guidance etc. |  |
| Evaluation | Evaluating the impacts of the policy |  |

**Table S2: Codes describing barriers and enablers to policy development and implementation**

| Code/theme | Description | Reference |  |
| --- | --- | --- | --- |
| External drivers | | |  |
| Events & disasters | Events/disasters that raise awareness of the problem | WHO report^2^ |  |
| Advocacy by interest groups | Groups external to government raising the issue for consideration within policy | WHO report^2^ |  |
| International agendas/protocols | International organisations promoting/highlighting issues (i.e., climate agreements) | WHO report^2^ |  |
| Economic/financial | | |  |
| Public funding | Funding for the policy | WHO report^2^ |  |
| Economic (dis)incentives | Financial benefits or drawbacks of the policy | WHO report^2^ |  |
| Collaborations | | |  |
| Cross-sectoral partnerships | Collaboration between housing and health professionals | WHO report^2^ |  |
| Collaborations between academics and policy makers/stakeholders | Academics working closely with policy makers | WHO report^2^ |  |
| Knowledge | | | |
| Scientific evidence | Scientific evidence on the problem | WHO report^2^ |  |
| Awareness of health and housing | Awareness/understanding of health and housing by stakeholders | WHO report^2^/ Carmichael^3^ |  |
| Awareness/interest in policy | Awareness of/Interest in a policy existence and its purpose | WHO report^2^ |  |
| Perception of policy outcomes | | | |
| Positive consequences | Positive consequences of the policies | WHO report^2^ |  |
| Negative consequences | Negative consequences of the policies | WHO report^2^ |  |
| Perceived consequences | Perceived consequences of policies outcomes | WHO report^2^ |  |
| Policy processes and resources | | |  |
| Enforcement systems | Enforcement procedures that support implementation/create accountability | WHO report^2^ |  |
| Personnel skills | Personnel skills for policy implementation, including trained and knowledgeable staff | WHO report^2^/  Weiss^4^ |  |
| Institutional capacity | Adequate institutional capacity/processes and systems | Carmichael^3^ |  |
| Clear tools and communication | clarity and usability of policy guidance, communication and tools | WHO report^2^ |  |
| Realistic time-frames |  | Weiss^4^ |  |

**References**

1. Howlett M, McConnell A, Perl A. Weaving the Fabric of Public Policies: Comparing and Integrating Contemporary Frameworks for the Study of Policy Processes. *J Comp Policy Anal Res Pract*. 2016;18(3):273-289. doi:10.1080/13876988.2015.1082261

2. Removed to be anonymous at review stage

3. Carmichael L, Barton H, Gray S, Lease H, Pilkington P. Integration of health into urban spatial planning through impact assessment: Identifying governance and policy barriers and facilitators. *Environ Impact Assess Rev*. 2012;32(1):187-194. doi:10.1016/j.eiar.2011.08.003

4. Weiss D, Lillefjell M, Magnus E. Facilitators for the development and implementation of health promoting policy and programs - A scoping review at the local community level. *BMC Public Health*. 2016;16(1). doi:10.1186/s12889-016-2811-9
